# *β*-Fructofuranosidase and *β* –D-Fructosyltransferase from New* Aspergillus carbonarius* PC-4 Strain Isolated from Canned Peach Syrup: Effect of Carbon and Nitrogen Sources on Enzyme Production

**DOI:** 10.1155/2019/6956202

**Published:** 2019-01-08

**Authors:** Gustavo Carvalho do Nascimento, Ryhára Dias Batista, Claudia Cristina Auler do Amaral Santos, Ezequiel Marcelino da Silva, Fabrício Coutinho de Paula, Danylo Bezerra Mendes, Deyla Paula de Oliveira, Alex Fernando de Almeida

**Affiliations:** ^1^Bioprocess Engineering and Biotechnology, Federal University of Tocantins, Gurupi, Tocantins, Brazil; ^2^Graduate Program on Food Science and Technology, Federal University of Tocantins, Palmas, Tocantins, Brazil; ^3^Graduate Program on Biotechnology and Biodiversity of Amazonian–Bionorte, Federal University of Tocantins, Tocantins, Brazil; ^4^Superintendência de Desenvolvimento Científico e Tecnológico, Fundação de Amparo à Pesquisa do Estado do Tocantins (FAPT), Tocantins, Brazil; ^5^Habite, Biotechnology-Based Companies Incubator, Federal University of Tocantins, Gurupi, Tocantins, Brazil

## Abstract

*β*-fructofuranosidase (invertase) and *β*-D-fructosyltransferase (FTase) are enzymes used in industrial processes to hydrolyze sucrose aiming to produce inverted sugar syrup or fructooligosaccharides. In this work, a black* Aspergillus *sp. PC-4 was selected among six filamentous fungi isolated from canned peach syrup which were initially screened for invertase production. Cultivations with pure carbon sources showed that invertase and FTase were produced from glucose and sucrose, but high levels were also obtained from raffinose and inulin. Pineapple crown was the best complex carbon source for invertase (6.71 U/mL after 3 days of cultivation) and FTase production (14.60 U/mL after 5 days of cultivation). Yeast extract and ammonium chloride nitrogen sources provided higher production of invertase (6.80 U/mL and 6.30 U/mL, respectively), whereas ammonium nitrate and soybean protein were the best nitrogen sources for FTase production (24.00 U/mL and 24.90 U/mL, respectively). Fermentation parameters for invertase using yeast extract were *Y*_*P*/*S*_ = 536.85 U/g and *P*_P_ = 1.49 U/g/h. FTase production showed values of *Y*_*P*/*S*_ = 2,627.93 U/g and *P*_*P*_ = 4.4 U/h using soybean protein. The screening for best culture conditions showed an increase of invertase production values by 5.10-fold after 96 h cultivation compared to initial experiments (fungi bioprospection), while FTase production increased by 14.60-fold (44.40 U/mL) after 168 h cultivation.* A. carbonarius* PC-4 is a new promising strain for invertase and FTase production from low cost carbon sources, whose synthesized enzymes are suitable for the production of inverted sugar, fructose syrups, and fructooligosaccharides.

## 1. Introduction

Invertases (1, 2-*β*-D-fructofuranosidase fructohydrolase, EC 3.2.1.26) are members of the GH32 family of glycoside hydrolases that catalyze the hydrolysis of *α*-1,4-glycosidic bonds from nonreducing fructofuranoside terminal residues of *β*-fructofuranosides (sucrose) to give an equimolar mixture of monosaccharide D-glucose and D-fructose, called invert sugar [1–3]. Aqueous solutions of both sucrose and glucose are slightly dextrorotatory, i.e., causing rotation of plane polarized light to right, while the solution of fructose is strongly levorotatory, i.e., causing rotation of plane polarized light to left [4]. Invertases are widely used in food and beverage industries. One of important applications of invertase is the production of noncrystallizable sugar syrup (invert sugar syrup) from sucrose. Invert sugar is used as a humectant in the manufacture of soft candies and fondants [5]. Invertase can be also used when sucrose containing substrates are subjected to fermentation, viz., production of alcoholic beverages, lactic acid, and glycerol. Furthermore, invertases are also used for the manufacture of artificial honey, plasticizing agents, and fructooligosaccharides [5].

Fructosyltransferase (FTase; EC 2.4.1.9) hydrolyzes sucrose and transfer fructosyl group to a sucrose molecule acceptor to generate fructooligosaccharides along with glucose and fructose, which have a wide range of applications in food and pharmaceutical industries [6, 7]. Fructooligosaccharides can be obtained synthetically from agave fructans by acid-catalyzed hydrolysis, although the formation of fructooligosaccharides via enzymatic methods is preferred due to high substrate specificity and selectivity of enzymes compared to chemical routes [8]. Fructooligosaccharides from sucrose are considered as new alternative sweeteners with functional properties, also called soluble fibres with several desirable characteristics such as low calories, noncariogenicity and safety for diabetics. Fructooligosaccharides are also known as prebiotics, since they stimulate the growth of probiotic organisms [1]. However, fructosyltransferase and fructofuranosidase production is rather confusing differing from one source to another, from one microorganism to another, and even from one strain to another [9]. In addition, invertases exhibit transfructosylating activity at high sucrose concentrations in order to release glucose and fructose from sucrose. Microbial invertases may catalyze the synthesis of short-chain fructooligosaccharides such as 1-kestose, nystose, and fructofuranosyl nystose, in which one to three fructosyl moieties are linked to the sucrose by different glycosidic bonds depending on the enzyme source [4, 5].

Invertases can be found by wide range of organisms including plants, animal, bacteria, yeasts, and filamentous fungi. Among filamentous fungi,* Aspergillus* genus has been extensively used to produce invertase under submerged and solid-state cultivations and its mechanisms of enzyme production have also been investigated.* Aspergillus niger *can produce invertase in the presence of *β*-fructofuranoside sugars, demonstrating that the synthesis of invertase is inducible [10]. This strain produces invertase with only hydrolytic activity, producing equimolar amounts of fructose and glucose. On the other hand, the FTase produced by this strain shows exclusively fructosyl transfer reaction producing glucose, 1-kestose, nystose, and fructofuranosyl nystose [9].* Aspergillus flavus* produced high yields of invertase under optimized conditions in submerged cultures [11]. Guimarães et al. [12] observed that* Aspergillus ochraceus* produced high levels of a thermostable extracellular invertase when cultured in Khanna medium supplemented with sugarcane bagasse as carbon source.

Enzyme production requires long time of laboratorial experiment to find the ideal conditions and cost-effectiveness for microbial cultivation. Submerged and solid-state cultivations are commonly employed to enzyme production using alternative substrates as an attempt to reduce bottlenecks involved in the initial steps of production, e.g., high costs of production using high value-added substrates. The utilization of renewable resources such as agroindustrial residues as cheap and readily available substrates for enzyme production has been a subject of intense research as an alternative for cost-effective enzyme production and waste management [2]. Agroindustrial residues are generated in large amounts every year and their usage for bioprocesses is particularly interesting due to their renewability, low-cost, and suitable characteristics which allow the production of different value-added metabolites [13]. Giraldo et al. [14] used agroindustrial byproducts, e.g., sugarcane bagasse, oat meal, cassava flour, corn cob, soy bran, and wheat bran for invertase production under submerged cultivation. Their experiments demonstrated that agroindustrial residues provided the production of higher levels of extracellular invertase by* Paecilomyces variotii*. Guimarães et al. [12] observed that highest levels of extracellular invertase activity were obtained when Khanna medium was supplemented by sugar cane bagasse as carbon source, and the extracellular invertase activity comprised 98% total enzyme activity (intracellular + extracellular) from cultivation. Oyedeji et al. [2] reported that agroindustrial residues are suitable substrates for microbial cultivation and consequent production of molecule of biotechnological interest such as enzymes and other value-added chemicals.

The aims of this work were to select an invertase-producing* Aspergillus* spp. strain with high fructosyltransferase activity from canned peach syrups under submerged cultivations. Nutritional parameters, such as pure and complex carbon sources and nitrogen sources, were evaluated to obtain high yield of enzymes. Agroindustrial residues were also evaluated as alternative substrates for enzyme production.

## 2. Material and Methods

### 2.1. Microorganisms

Filamentous fungi were isolated from a jar surface containing canned peach syrup. Canned peach syrup was obtained from local market. Samples of canned peach syrup were maintained at 30°C for five days. Colonies grown on the surface of syrup were transferred to Petri plates containing potato dextrose agar (PDA) and incubated at 30°C for five days. Isolated colonies were inoculated at the center of the Petri dishes to ensure the purity of fungi strains. Filamentous fungi preservation was conducted in sterile distilled water [15]. Dishes (approximately 5 mm) containing small portion of culture medium, mycelium, and spores were aseptically transferred into 6 mL sterile antibiotic flasks, which were added to 4 mL sterile distilled water and sealed with rubber stopper. Castellani flasks were kept at 4°C and the viability of strains was verified periodically every six months.

Fungi were isolated as monocultures on two media: malt extract agar and potato dextrose agar. Fungi were identified to genus level according to morphological structures [16–18].

### 2.2. Inoculum Preparation

Dishes containing mycelium and spores were aseptically inoculated in PDA plates and incubated at 30°C for five days. Filamentous fungi colonies were transferred to PDA slants and incubated at 30°C for five days. The spore suspension was carried out using sterile NaCl 0.85% (w/v). Culture media for screening of enzyme production were inoculated with 1 mL of 10^6^ spores/mL suspension.

### 2.3. Culture Conditions for Enzyme Production

Filamentous fungi cultures were performed in Erlenmeyer flasks (125 mL) containing 20 mL of Vogel medium solution [19]. A stock medium 50-fold concentrated was prepared containing (g/L): sodium citrate*∗*5H_2_O, 150; KH_2_PO_4_, 250; MgSO_4_*∗*7H_2_O, 10; CaCl_2_*∗*2H_2_O, 5; trace elements solution, 5 mL stock solution (g/L): citric acid*∗*H_2_O, 50; ZnSO_4_*∗*7H_2_O, 50; Fe(NH_4_)_2_(SO_4_)_2_*∗*6H_2_O, 10; CuSO_4_*∗*5H_2_O, 2.5; MnSO_4_*∗*H_2_O, 0.05; H_3_BO_3_, 0.05; Na_2_MoO_4_*∗*2H_2_O, 0,05; biotin solution (0.1 mg/mL), 5 mL. Chloroform (0.2 mL) was added as a preservative. The solutions were maintained at 4°C. Medium preparation consisted of 50-fold dilution of stock medium, supplemented with carbon sources (1%, w/v). Yeast extract (0.2%, w/v) was used as nitrogen source. The final pH was adjusted to 6.0. Flasks containing culture media were sterilized in autoclave at 121°C for 15 min, inoculated with spore suspension previously prepared, and incubated in orbital shaker for 72 and/or 120 hours, at 28°C, 180 rpm.

### 2.4. Crude Extract Preparation

Biomass was separated from fermentation broth by filtration (cellulose acetate membrane 0.45 *μ*m) and dried at 105°C until constant weight. Cell free broth (crude extract) was used for enzyme activity assays.

### 2.5. Bioprospecting of Filamentous Fungi

Filamentous fungi were screened for invertase production using Vogel's medium supplemented with sucrose as sole carbon source (1.0%. w/v) and 0.2% (w/v) yeast extract as nitrogen source. The cultures were carried out in Erlenmeyer flasks (125 ml) containing 20 mL medium and pH was adjusted to 6.0. The cultures were incubated at 180 rpm and 28°C for 120 hours. All experiments were performed in triplicate.

### 2.6. Molecular Identification of* Aspergillus *sp. PC-4

Total DNA from filamentous fungal strains was extracted from PDA growth medium using IllustraTM Nucleon PhytoPure, plant and fungal DNA extraction kits (GE Healthcare, Amersham, England). DNA was quantified using NanoDrop 2000 spectrophotometer (Thermo Scientific, Uniscience, Brazil) and then diluted to a concentration of 50 ng for polymerase chain reaction (PCR) using the primers ITS1 (5′-TCCGTAGGTGAACCTGCGG-3′) and ITS4 (5′-TCCTCCGCTTATTGATATGC-3′) [20]. Amplifications were performed on Mastercycler® nexus (Eppendorf) thermocycler using the GoTaq® DNA Polymerase kit (Promega Madison-USA) with final reaction volume of 25 *μ*L. Thereafter, PCR products were checked by 1% (w / v) agarose gel electrophoresis [21] and visualized under ultraviolet light on LPIX-EX photodocumentator (Loccus Biotechnology São Paulo, Brazil). The 1 Kb DNA Ladder (Promega Canada, USA) was used as molecular weight marker. The amplified products were purified with Exonuclease I (ExoSap-IT®) and Alkaline Phosphatase solution (USB Corporation Austin-USA) followed by sequencing using the same PCR primers on ABI 3500 xl automatic sequencer (Life Technologies) according to the Dideoxy or chain termination method [22] using the BigDye Terminator v 3.1 sequencing kit (Life Technologies Foster city, USA). This stage was carried out at the Laboratory of Polar Microbiology and Tropical Connections of Federal University of Minas Gerais (UFMG) and Laboratory of Cellular and Molecular Parasitology (LCPM) of Oswaldo Cruz Foundation-Fiocruz/René Rachou Institute.

### 2.7. Phylogenetic Analysis

The sequence data were obtained from NCBI Genbank database available under* Aspergillus carbonarius* with accession n^o^ AJ876878 and were used for comparisons with the 599 bp from our isolate. Sequences with high query coverage and homology were selected for phylogenetic analysis using the neighbor-joining method [23]. The sequences were aligned using software Clustal W analysis [24], and the phylogenetic analyses were performed using MEGA 6.0 for neighbor-joining and bootstrap analysis. Sequence from this study was indicated in the tree by collection code, while GenBank sequences were indicated by accession numbers.

### 2.8. Effect of Pure and Complex Carbon Sources

Pure carbon sources (fructose, glucose, raffinose, cellulose, cellobiose, sorbitol, inulin, sucrose, maltose, lactose, and xylose) were used as sole carbon sources. Complex carbon sources (pineapple crown, sweet potato flour, sugarcane bagasse, corn cob, cassava peel, soybean peel, soybean bran, corn straw, bacaba peel, sorghum bran, and wheat bran) were used as sole carbon sources, which were previously washed with distilled water, dried until constant weight, and sieved (18 mesh). The cultures were carried out in Erlenmeyer flasks (125 ml) containing 20 mL medium, supplemented with 1.0% (w/v) pure or complex carbon sources, 0.2% (w/v) yeast extract as nitrogen source, and pH was adjusted to 6.0. The cultures were incubated at 180 rpm and 28°C for 72 h for invertase production and 120 h for FTase production. Cultures were carried out in triplicate.

### 2.9. Effect of Nitrogen Sources

Nitrogen sources (ammonium nitrate, ammonium chloride, ammonium sulfate, yeast extract, peptone, soybean protein, and corn steep liquor) were used as sole inorganic or organic sources. The cultures were prepared in Erlenmeyer flasks (125 ml) containing 20 mL of medium, supplemented with 0.2% (w/v) inorganic or organic source individually, 1.0% (w/v) pineapple peel as carbon source, with pH adjusted to 6.0. The cultures were incubated at 180 rpm and 28°C for 72 h for invertase production and 120 h for FTase production.

### 2.10. Time-Course of Enzyme Production

Time-course of invertase production was carried out in Erlenmeyer flasks (125 mL) containing 20 mL of Vogel's medium supplemented with 1% pineapple peel, 0.2% yeast extract, and pH 6.0. The cultures were incubated at 180 rpm and 28°C for 120 h. Samples were withdrawing each 12 h of cultivation.

Time-course of FTase production was carried out in Erlenmeyer flasks (125 mL) containing 20 mL of Vogel's medium supplemented with 1% pineapple peel, 0.2% ammonium chloride, and pH adjusted to 6.0. The cultures were incubated at 180 rpm, 28°C for 240 hours. Samples were periodically analyzed at intervals of 12 h cultivation.

### 2.11. Biomass Characterization

Total organic carbon was carried out by Walkley-Black method [25]. Total carbohydrate was determined by differences among moisture, gray, protein, and lipid [26]. Total nitrogen was determined by micro-Kjeldahl procedures [27].

### 2.12. Enzyme Assays

Invertase activity was assayed by measuring the amount of reducing sugars from sucrose. Eight hundred microliter of sucrose (2% w/v) diluted in McIlvaine buffer solution pH 5.0 were dispensed into test tube and kept at 50°C for 5 min. Then, 200 *μ*L of crude extract were added to this reaction medium. The reaction was stopped in different time intervals (0, 5 and 10 min) by taking 200 *μ*L reaction medium and dispensed into another test tube containing 200 *μ*L of 3,5-dinitrosalicylic acid. Reducing sugars were estimated colorimetrically with 3,5-dinitrosalicylic acid [28] using fructose as standards. One unit of enzymatic activity is defined as the amount of enzyme that releases 1 *μ*mol of reducing sugars per minute per milliliter.

FTase activity was determined by incubating 100 *μ*L culture filtrate with 400 *μ*L sucrose (20% w/v in 0.1 M sodium acetate buffer pH 5.0) at 50°C for 1 h. The reaction was stopped by boiling the mixture at 100°C for 10 min. FTase activity was estimated by adding 10 *μ*L of appropriately diluted reaction to 1 ml test reagent (glucose oxidase–peroxidase, GOPOD kit, Sigma). The glucose released was measured at 510 nm. One unit of FTase was defined as the amount of enzyme required to produce 1 *μ*mol of glucose per minute under assayed conditions.

### 2.13. Fermentation Parameters

Enzyme yield (Y, unit of enzyme/g of carbon source) is

(1) $$\begin{array}{c} Y_{\left(P/S\right)}=\frac{P_{f}-P_{0}}{S_{0}} \end{array}$$

where *P*_*f*_ is total enzyme produced after fermentation; *P*_0_ is total enzyme in initial fermentation; *S*_0_ is substrate concentration in initial fermentation.

Productivity assay (P, unit of enzyme/time of cultivation) is

(2) $$\begin{array}{c} P_{P}=\frac{E_{t}}{t} \end{array}$$

where *E*_*t*_ is total enzyme produced after fermentation;* t* is time of the fermentation process.

### 2.14. Statistical Analysis

The results were expressed as mean ± standard error of the mean and subjected to analysis of variance (ANOVA), followed by Tukey test.

## 3. Results and Discussion

Initially, filamentous fungi were isolated from canned peach syrup and cultivated in submerged cultures containing sucrose as sole carbon source (Table 1). Among six isolates identified as* Aspergillus* sp. and* Penicillin *sp., the PC-4 strain presented the highest invertase production (2.47 U/mL). Fermentation parameters also demonstrated that* Aspergillus *sp. PC-4 can be a promising invertase producer, whose production yield from sucrose was 222.30 U/g and the productivity was 0.37 U/h. The highest biomass production was observed for PC-6 (6.02 g/L) followed by* Aspergillus *sp. PC-1, PC-2, PC-4, and PC-5 (5.72 g/L, 5.56 g/L, 5.54 g/L, and 5.37 g/L, respectively).* Aspergillus *genus has been highlighted as an invertase producer under both submerged and solid-state cultivations [29]. Veana et al. [3] related invertase production by xerophilic* A. niger* under submerged cultivation among eight strains evaluated only* A. niger *GH1 produced 8.6 U/mL invertase after 48 h cultivation. In the present study, PC-5 and PC-1 strains produced, respectively, 1.96 and 1.86 U/mL invertase, followed by PC-6, PC-2 (1.30 U/mL, 1.23 U/mL, respectively).

*Penicillium *sp. PC-3 showed invertase production of 1.06 U/mL and production yield of 95.20 U/g and productivity of 0.16 U/h.* Penicillium* genus was also reported in the literature as invertase producer and its enzyme has potential for the production of inverted sugar and fructooligosaccharides. Poonawalla et al. [30] related that maximum invertase production by* Penicillium chrysogenum* was intracellular and maximum extracellular invertase was reached after cell autolysis. On the other hand,* Penicillium purpurogenum*,* Penicillium pinophilum*, and* Penicillium citrinum* produced extracellular invertase under submerged conditions from sucrose as carbon source [3]. Invertase from* Penicillium expansum* was also produced under submerged conditions using cells immobilized in synthetic fibers and polyurethane foam with fructooligosaccharides production being simultaneously evaluated along enzyme production, with fructooligosaccharides production of 120.3 and 104.8 g/L, respectively, and high invertase activity (23.01 U/mL and 32.42 U/mL, respectively) [31].

For the next step of this work,* Aspergillus* sp. PC-4 was selected to parametric optimization of culture medium for *β*-fructofuranosidase and *β* –D-fructosyltransferase production under submerged conditions. Although low invertase production values have been obtained from other screened fungi, it was clear that these fungal strains are potential enzyme producers for food industry and their cultivation parameters demand future investigation.

### 3.1. Molecular Identification of Aspergillus sp. PC-4

**Figure 1:**
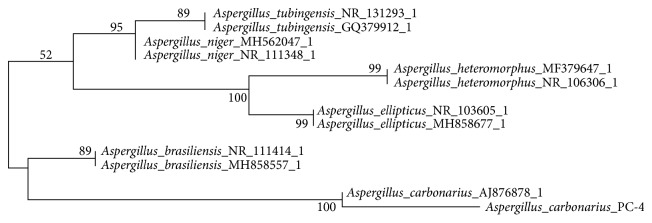
The neighborjoining tree inferred from the partial sequence of the ITS region dataset using Mega 6.0. The bootstrap was 2000 replications to assess the reliable level to the nods of the tree.

Molecular identification revealed that the isolated strains showed 100% sequence similarity with* Aspergillus carbonarius* (GeneBank accession n^o^ AJ876878) (Figure 1). Therefore, the isolated strain was referred to in this work as* Aspergillus carbonarius *PC-4.* A. carbonarius *strains have been extensively studied for ochratoxin A production in fruits and beverages [32–34]. Regarding enzyme production,* A. carbonarius *have been studied for cell plant polysaccharides degradation [35], polygalacturonase and pectinase production [36–38], and also *β*-Xylosidase [39], among other biotechnological studies. To our knowledge, in this study* A. carbonarius *PC-4 strain was first time reported to produce invertase and fructosyltransferase under cost-effective fermentation media.

### 3.2. Effect of Pure Carbon Sources on Invertase and FTase Production

**Figure 2:**
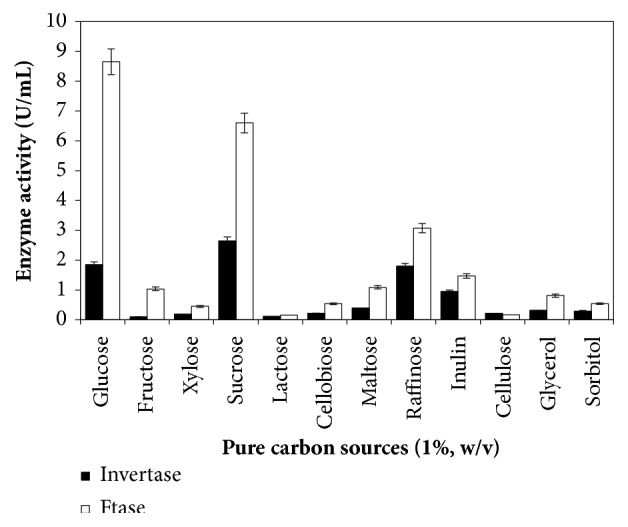
Pure carbons sources on invertase and *β*-D-fructosyltransferase production by black* Aspergillus *sp. PC-4 strain. Culture conditions: cultivation was carried out at 28°C, 180 rpm for 120 hours.

In order to determine the regulatory mechanism of invertase and FTase synthesis by* A. carbonarius* PC-4, several carbohydrates, besides glycerol and sorbitol, were used as sole carbon sources in the cultures (Figure 2). In these experiments, mono-, di-, and poly-carbohydrate and alcohol were evaluated as energy and inducing carbon source to distinguish the production mechanism and secretion of enzymes. Inducible enzymes are produced in the presence of a potential substrate which stimulates the enzyme production and secretion, whereas the constitutive enzymes can be produced in the absence of inducing substrate and this carbon source can be used as adjunct to increase the enzyme production.

The highest invertase production was observed from sucrose (2.65 U/mL), whereas highest FTase production was obtained from glucose (8.65 U/mL). Glucose and raffinose were intermediary sources for invertase production (1.85 U/mL and 1.80 U/mL, respectively). Sucrose and raffinose were also intermediary sources to FTase production (6.60 U/mL and 3.07 U/mL, respectively). The lowest enzyme production values were observed using fructose, lactose, cellobiose, maltose, inulin, cellulose, glycerol, and sorbitol.

Fermentation parameters showed highest invertase yield of 265.0 U/g and productivity of 0.73 U/h from sucrose, followed by glucose (*Y*_*P*/*S*_ = 185.0 U/g; *P*_*P*_ = 0.51 U/h) and raffinose (*Y*_*P*/*S*_ = 180.0 U/g; *P*_*P*_ = 0.50 U/h). Regarding FTase production, the highest fermentation parameter values were observed from glucose (*Y*_*P*/*S*_ = 865.0 U/g; *P*_*P*_ = 2.40 U/h), followed by sucrose (*Y*_*P*/*S*_ = 660.0 U/g; *P*_*P*_ = 1.83 U/h) (Table 2). Biomass produced by filamentous fungi from pure carbon source ranged from 3.81 g/L to 6.72 g/L, with sucrose and glucose resulting in 6.31 g/L and 6.41 g/L biomass, respectively. Raffinose was observed as pure carbon sources for invertase production using* Paecilomyces variotii *[14],* Aspergillus nidulans,* and* Aspergillus fumigatus *[40]. Rezende and Felix [40] related that raffinose as well as other oligosaccharides may be hydrolyzed by *α*-galactosidase or invertase (*β*-fructofuranosidase) which cleaves the *α*-1,2 linkage of sucrose moiety producing melibiose and fructose. Vainstein and Peberdy [41] observed that invertase activities were found in* A. nidulans* cultures using sucrose and raffinose as the best inducers, and raffinose seemed to be the best carbon source for invertase secretion.

Our results suggest that* A. carbonarius* PC-4 produces a constitutive enzyme using a wide variety of carbohydrates and glycerol alcohol. Vainstein and Peberdy [41] related that the* A. nidulans *produced a constitutive level of invertase in a wide variety of carbohydrates with 3% glucose resulting in the lowest activity, while 1% raffinose and sucrose provided highest activities. In this study, maltose, melibiose, glycerol, and trehalose (1% w/v) also induced invertase production at similar values. Rubio and Navarro [10] evaluated the regulation of invertase production by* Aspergillus niger *strain isolated from rotten lemons. The highest invertase productions were observed from inulin, raffinose, turanose, and sucrose. The authors suggest that inducing sugars of invertase synthesis exhibit the *β* linkage and fructose located at the end of molecule as a common feature of their structure. The *β* linkage does not cause induction of enzyme production, as shown with cellobiose and gentiobiose, whereas the *α* linkage has not effect at all, as demonstrated with maltose and melibiose. On the other hand, glucose and fructose failed to induce the invertase synthesis, a fact that suggests these carbohydrates are not involved in the mechanism of invertase synthesis induction for* A. niger *strain.

Glucose has extensively been related as repressor of invertase production by many filamentous fungi [14, 42, 43]. Glucose supplementation (1.0%, w/v) was the second carbon source which induced more efficiently the invertase production by* A. carbonarius* PC-4 (Figure 2 and Table 2). Alegre et al. [44] observed an intracellular invertase level 5-fold higher from 1% glucose than the absence of this carbon source in cultivations of* Aspergillus caespitosus*. However, all glucose concentrations were tested, with exception of 1.0% and 2.0% glucose (w/v); the extracellular activity was higher than the intracellular one. Giraldo et al. [14] reported the effect of glucose or fructose addition to the culture media containing soy bran as main carbon source over intra- and extracellular invertase production by* P. variotii* under submerged fermentation.

### 3.3. Effect of Complex Carbon Sources on Invertase and FTase Production

The major bottleneck in enzyme production and its industrial application is the relative high production cost and low enzyme yields. The use of agroindustrial residues as raw materials decreases the impact on environment, since the reuse of waste materials can reduce their accumulation in the environment with additional economic value due to their biotechnological application [45]. Besides, agroindustrial residues can be used as carbon sources by several microorganisms to produce high-value added chemicals such as carbohydrases, including invertases and fructosyltransferases. Recent studies have focused on the use of agroindustrial residues for industrial enzyme production, since it is one of the possible ways to reduce the production costs significantly [46].

**Figure 3:**
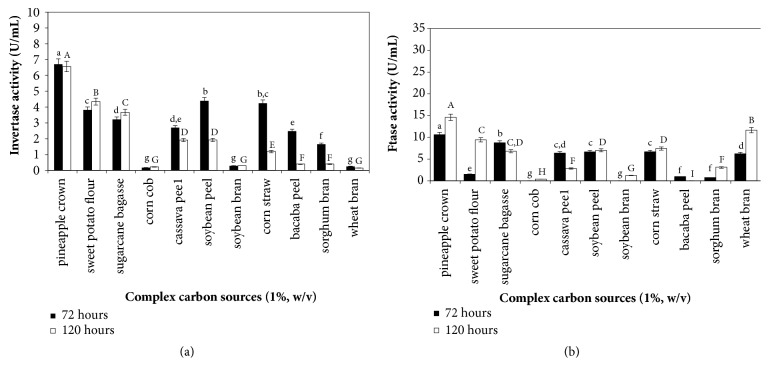
Complex carbon sources on invertase (a) and *β*-D-fructosyltransferase (b) by black* Aspergillus* sp. PC-4. Culture conditions: complex carbon sources (1.0%, w/v), yeast extract (0.2% w/v), 28°C, 180 rpm. Mean values with the same letter do not statistically differ from each other by the ANOVA Tukey test (p = 0.05).

In this study, eleven agroindustrial residues were used as carbon sources for invertase and FTase production by* A. carbonarius* PC-4 (Figure 3). The invertase and FTase production presented significance (p<0.05) when pineapple crown was used as carbons sources. The highest invertase activity (6.71 U/mL) was observed after 3 days of cultivation; and highest FTase activity was observed after 5 days of cultivation (14.60 U/mL), representing 1.45-fold higher than values obtained from other tested carbon sources.

Fermentation parameters showed the highest values for invertase production (*Y*_*p*/*s*_ = 586.97 U/g of substrate and *P*_*p*_ = 0.98 U/h) and FTase (*Y*_*p*/*s*_ = 1,459.00 U/g of substrate and *P*_*p*_ = 1.94 U/h) after 3 and 5 days of cultivations, respectively, using pineapple crown as carbon source (Table 3). Oyedeji et al. [2] observed invertase production of 21.46 U/mL after 120 h incubation using pineapple peel as substrate. These authors stated that the increased invertase production might be due to utilization of pineapple peel by* A. niger* IBK1 for growth and hence enzyme production. However, decreasing values of enzyme production could be due to exhaustion of glucose, a primary metabolite obtained in the degradation of substrate, which is required for growth or increase of metabolic intermediates secreted into fermentation medium.

In this study, pineapple crown was chemical characterized presenting 28.6% total carbon, 1.8% total nitrogen, with a C/N ratio of 15.7, 7.7% humidity, 6.0% ashes, 6.7% total lipids, t11.5% total protein, and 68.1% total carbohydrates. Pineapple wastes were also used to produce other carbohydrate hydrolases, such as *β*-glucosidase [46] and cellulases [47]. Pineapple peel is a byproduct resulted from canning processing of pineapple which produces about 35% fruit waste and lead to serious environmental pollution. This waste contains considerable amounts of soluble sugars such as sucrose which makes it suitable as substrate for microbial fermentations [2, 48]. Therefore, the pineapple peel composition is an attractive alternative for fungal growth due to its low cost and availability of carbon and nitrogen sources beside minerals. Agroindustrial residues such as pineapple peel are therefore suitable substrates for microbial cultivation and production of molecules with biotechnological importance such as enzymes and other value-added products [49]. On the other hand, pineapple crown is firstly related in this study as a potential carbon source substrate for enzyme production and to obtain value-added products. Pineapple crown used for invertase and FTase production presented centesimal composition as follows (%): moisture, 7.70 ± 0.75; lipids, 6.70 ± 1.32; proteins, 11.48 ± 0.22; carbohydrates, 68.10 ± 1.03; ashes, 6.05 ± 0.07; carbon, 28.65 ± 0.18; and nitrogen, 1.84 ± 0.03.

Intermediary values of invertase production were found with soybean peel and corn straw (4.39 U/mL and 4.24 U/mL, respectively) after 3 days of cultivation and sweet potato flour (4.35 U/mL) after 4 days of cultivation. Sugarcane bagasse, cassava peel, and bacaba peel can be considered substrates with potential use for invertase production after optimization process of culture conditions since these substrates induced the enzyme production ranging from 2.5 to 3.22 U/mL, whereas wheat bran, soybean bran, sorghum bran, and corn cob were weak inducers for invertase production by black* A. carbonarius* PC-4. On the other hand, intermediary values of FTase production were observed from cassava peel, soybean peel, corn straw, sugarcane bagasse, sweet potato, and wheat bran (6.40 to 11.65 U/mL); and low production was observed from corn cob, soybean bran, bacaba peel, and sorghum bran. The fermentation parameters using sweet potato flour, sugarcane bagasse, corn cob, and wheat bran can indicate that these substrates would be potential carbon sources for invertase and FTase production under optimized conditions or used as mixed substrates in fermentation processes. Guimarães et al. [12] stated that many enzymes of industrial significance are regulated by carbon source composition, and the use of agroindustrial residues for extracellular invertase production by* Aspergillus ochraceus *using sugarcane bagasse and corn cob was effective in promoting the production of extracellular invertase, substantially lowering the production costs. Giraldo et al. [14] have reported the promising utilization of liquid medium supplemented with agroindustrial wastes as carbon source, especially soy bran or wheat bran, resulting in higher extracellular activity of invertase produced by* Paecilomyces variotii*, which was 7 and 46 times higher from soy bran than those estimated for sucrose and glucose, respectively.

### 3.4. Effect of Nitrogen Sources on Invertase and FTase Production

**Figure 4:**
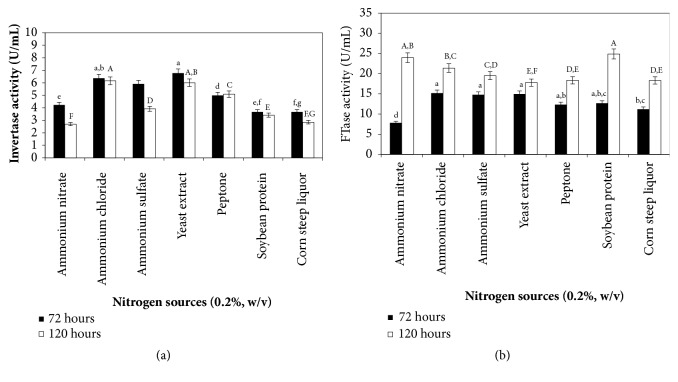
Organic and inorganic nitrogen sources on invertase (a) and *β*-D-fructosyltransferase (b) production by* A. carbonarius* PC-4. Culture conditions: pineapple crown (1.0%, w/v), nitrogen sources (0.2% w/v), 28°C, 180 rpm. Mean values with the same letter do not statistically differ from each other by the ANOVA Tukey test (p = 0.05).

Nitrogen sources have an important role in fermentation processes, in which the microbial growth and enzyme secretion are directly affected by nitrogen composition. Organic and inorganic nitrogen sources play an important role in the synthesis of enzymes, since inorganic nitrogen sources can be used quickly, and organic sources can supply many cell growth factors and amino acids needed for cell metabolism and enzyme production [50]. The invertase and FTase production by* A. carbonarius* PC-4 using different nitrogen sources such as ammonium ions (ammonium nitrate, ammonium chloride and ammonium sulfate), yeast extract, peptone, soybean protein, and corn steep liquor after three and five days of cultivation can be observed in Figure 4. Highest invertase activities were observed from ammonium chloride and yeast extract (6.40 U/mL and 6.13 U/mL, respectively), which did not present differences in analysis of variance (p>0.05), followed by ammonium sulfate and peptone (5.90 U/mL and 5.50 U/mL, respectively) after three days of cultivation. Ammonium nitrate, soybean protein, and corn steep liquor induced the lowest invertase production. After five days of cultivation, the invertase activity decreased from all evaluated nitrogen sources, indicating a microbial growth suppression caused by exhaustion of nutrient in the culture medium.

Fermentation parameters for invertase production showed highest yield and productivity from yeast extract (*Y*_*P*/*S*_ = 536.85 U/g; *P*_P_ = 1.49 U/h) followed by ammonium sulfate (*Y*_*P*/*S*_ = 514.57 U/g; *P*_*P*_ = 1.37 U/h) after three days of cultivation (Table 3). The maximum biomass production was observed after three days of cultivation using organic or inorganic nitrogen sources (7.70 –8.53 g/L). After five days of cultivation biomass production decreased though FTase production was observed, suggesting an extracellular enzyme production which was secreted to the medium (Figure 3). Giraldo et al. [14] also observed the influence of organic and inorganic nitrogen sources on enzyme production. Maximal production of extracellular invertase was obtained when peptone was added to the culture medium, while peptone and ammonium phosphate were both the best nitrogen sources for the intracellular form. Vandáková et al. [6] observed similar results using yeast extract as nitrogen source. These authors related that the advantage of using yeast extract compared to inorganic nitrogen substrates possibly is based on supplementation of some essential growth factors for the microorganism.

FTase production was not statistically significant (p>0.05) for enzyme activity using inorganic and organic nitrogen sources although the highest activity was observed with soybean protein and ammonium nitrate (24.90 U/mL and 24.00 U/mL, respectively) after five days of cultivation. Fermentation parameters for FTase production under these evaluated conditions were *Y*_*P*/*S*_ = 2,627.93 U/g and *P*_*P*_ = 4.40 U/h for soybean protein and *Y*_*P*/*S*_ = 2,407.86 U/g and *P*_*P*_ = 4.00 U/h for ammonium nitrate. Ammonium chloride was also a promising nitrogen source for enzyme production, from which were obtained high invertase production value (21.40 U/mL) and significant fermentation parameters of yield and productivity (*Y*_*P*/*S*_ = 2,114.51 U/g and *P*_*P*_ = 3.12 U/h, respectively). The lowest FTase production was observed using yeast extract (17.80 U/mL, *Y*_*P*/*S*_ = 1,799.10 U/g and *P*_*P*_ = 3.00 U/h, respectively). FTase production after three days of cultivation showed similar activities among corn steep liquor, peptone, soybean protein, ammonium sulfate, yeast extract, and ammonium chloride ranging from 11.20 to 15.20 U/mL, except for ammonium nitrate from which was observed to have lower FTase activity of 7.80 U/mL. Under these conditions, productivity after three days of cultivations showed higher enzyme activity values compared to those obtained after five days, indicating which the culture parameters should be optimized to improve the enzyme production.

### 3.5. Time-Course of Invertase and FTase Production

**Figure 5:**
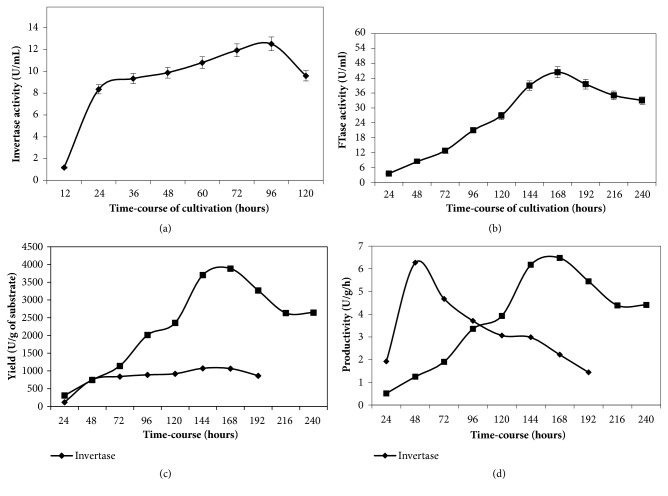
Time-course and fermentation parameters of invertase and *β*-D-fructosyltransferase (FTase) produced by *A. carbonarius*. PC-4. Legend: (a) invertase production; (b) FTase production; (c) yield of production for invertase and FTase; (d) productivity for invertase and FTase. Culture conditions: invertase production was carried out using pineapple crown (1.0%, w/v), yeast extract (0.2% w/v); FTase production was carried out using pineapple crown (1.0%, w/v) and soybean protein (0.2% w/v); cultures were carried out at 28°C, pH 6.0, and 180 rpm.

Time-course of invertase and FTase production by* A. carbonarius* PC-4 were carried out using the culture conditions established previously using the carbon and nitrogen sources selected (Figure 5). Invertase production was carried out using pineapple crown (1.0% w/v) as carbon source and ammonium chloride (0.2% w/v) as nitrogen source, while FTase production was carried out using pineapple crown (1.0% w/v) as carbon source and soybean protein (0.2% w/v) as nitrogen source. Both cultivations were carried out at 180 rpm and 28°C during 120 h and 240 h, respectively. Maximum invertase production was observed among 60 h and 96 h of cultivation (11.00 and 12.52 U/mL, respectively) with the highest production after 96 h (12.52 U/mL) (Figure 4(a)). Under these culture conditions, fermentation parameters showed maximum yield after 72 h with 1,073.90 U/g and productivity of 6.30 U/h after 24 h (Figures 4(c) and 4(d)). For FTase, the maximum production was observed after 168 h (44.40 U/mL) (Figure 4(b)). Fermentation parameters showed also maximum yield and productivity (*Y*_*P*/*S*_ = 5,982.71 U/g and *P*_*P*_ = 10.00 U/h, respectively) after 168 h.

## 4. Concluding Remarks

The screening of filamentous fungi from canned peach syrup presented potential* Aspergillus* spp. for invertase production under submerged fermentation. Among six potential isolates, black* Aspergillus* sp. PC-4 strain was selected to invertase and FTase production. This strain was identified as* Aspergillus carbonarius *PC-4. To our knowledge, it is the first time that* A. carbonarius *strain is reported as an invertase and FTase producer in the literature. Cultivation using pure carbon sources showed that this strain produces invertase in a constitutive way and FTase was inducible. Agroindustrial wastes were used as complex carbon sources and the pineapple crown was a promising substrate for invertase and FTase production. Furthermore, pineapple crown is firstly related in this study as carbon source for enzyme production. Nitrogen sources showed that organic and inorganic sources can be used to both enzymes' production though the ammonium chloride and yeast extract were the best nitrogen sources for invertase production (~6.6 U/mL) after three days of cultivation, while soybean protein and ammonium nitrate were the best sources for FTase production (~24 U/mL) after five days of cultivation. Time-course of enzyme production showed an increase of 5.10-fold in the invertase production compared to initial experiments (screening of fungi); and FTase production increased 3.0-fold after 196 h of cultivation compared to complex carbon sources (14.60 U/mL). This study showed that* A. carbonarius* PC-4 is a promising fungal strain for invertase and FTase production considering cost-benefit conditions and the enzymes produced are suitable for food and nutraceuticals industries in order to obtain inverted sugar, fructose syrups, and fructooligosaccharides.

## Figures and Tables

**Table 1:** Filamentous fungi strains morphologies isolated from canned peach syrups and invertase production.

Filamentous fungi	Colony morphology	Invertase activity	Yp/s	Pp	Biomass
		(U/ml)^*∗*^			(g/L)
*Aspergillus *sp. PC-1	Colonies are compact, yellow mycelium white with dense layer of black conidia heads. Conidial heads are globose, dark brown. Conidia are globose, dark brown.	1.86^b^	163.17	0.27	5.72
*Aspergillus *sp. PC-2	Colonies are granular, white mycelium with yellow-green conidia heads. Conidial heads are radiate. Conidia are globose, pale green.	1.23^c^	110.90	0.18	5.56
*Penicillium *sp. PC-3	Colonies are compact, white mycelium with dense layer of green conidia. Conidia are hyaline and in long chain, globose, greenish.	1.06^c^	95.20	0.16	2.67
*Aspergillus *sp. PC-4	Colonies are compact, yellow mycelium with dense layer of black conidia heads. Conidial heads are globose, dark brown. Conidia are globose, dark brown.	2.47^a^	222.30	0.37	5.54
*Aspergillus *sp. PC-5	Colonies are floccose, white-yellow mycelium, with brown conidia heads. Conidial heads are radiate. Conidia ares spherical, pale brown.	1.96^b^	151.82	0.25	5.37
*Aspergillus *sp. PC-6	Colonies are compact, white-yellow mycelium with dense layer of black conidia heads. Conidial heads are globose, dark brown. Conidia are globose, dark brown.	1.30^c^	97.28	0.16	6.02

Culture conditions: Cultivation were carried out at 28°C, 180 rpm for 72 hours. *∗* Mean values with the same letter do not statistically differ from each other by the ANOVA Tukey test (p = 0.05).

**Table 2:** Fermentation parameters for invertase and FTase production by *A. carbonarius* PC-4 on pure carbon sources.

Pure Carbon Sources	Fermentation Parameters				
	Invertase		Fructosyltransferase		Biomass
	Yp/s	Pp	Yp/s	Pp	(g/L)
Glucose	47.75	0.08	98.40	0.16	5.61
Frutose	9.93	0.02	29.99	0.05	5.27
Xylose	10.91	0.02	39.24	0.07	6.02
Sucrose	17.47	0.03	31.99	0.05	6.79
Lactose	13.27	0.02	12.36	0.02	6.30
Cellobiose	12.80	0.02	45.98	0.08	6.21
Maltose	19.11	0.03	87.10	0.15	6.27
Raffinose	100.51	0.16	145.70	0.24	4.51
Inulin	75.97	0.13	143.34	0.24	6.68
Cellulose	23.80	0.04	9.84	0.02	9.51
Glycerol	27.37	0.04	65.83	0.11	3.64
Sorbitol	14.59	0.02	45.98	0.08	6.56

Culture conditions: Cultivation were carried out at 28°C, 180 rpm, 72 hours.

**Table 3:** Fermentation parameters for invertase and *β*-D-fructosyltransferase production by black *Aspergillus *sp. PC-4 on complex carbon sources.

Component	Fermentation parameters									
	72 hours					120 hours				
	Invertase		FTase		Biomass	Invertase		FTase		Biomass
	Yp/s	Pp	Yp/s	Pp		Yp/s	Pp	Yp/s	Pp	
**Complex carbon sources (1.0**%**, w/v)**										
Pineapple crown	586.97	0.98	1,338.43	2.23	6.70	574.16	0.96	1,459.00	1.94	7.88
Sweet potato flour	352.80	0.59	331.43	0.55	5.24	402.21	0.67	730.35	1.21	5.76
Sugarcane bagasse	381.38	0.64	1,240.01	2.10	9.10	107.92	0.18	731.00	1.21	9.93
Corn cob	263.24	0.44	n.d	n.d	9.04	187.03	0.31	723.90	1.20	9.08
Cassava peel	131.98	0.22	402.72	1.42	6.78	33.10	0.06	248.32	0.41	6.34
Soybean peel	26.12	0.04	10.41	0.02	9.67	27.15	0.05	102.76	0.06	10.57
Soybean bran	273.52	0.46	n.d.	n.d.	5.61	312.26	0.52	476.18	0.79	5.21
Bacaba peel	8.40	0.01	5.82	0.27	9.80	21.51	0.04	n.d.	n.d.	9.99
Corn straw	22.68	0.04	24.76	0.04	10.98	13.86	0.02	40.62	0.06	11.63
Sorghum bran	229.13	0.38	282.02	0.50	5.71	37.91	0.06	252.40	0.42	6.48
Wheat bram	405.75	0.68	517.71	0.86	6.47	178.85	0.30	670.15	1.11	6.78

**Nitrogen sources (0.2**%**, w/v)**										
Ammonium nitrate	340.82	0.94	1,082.47	3.00	7.72	231.67	0.64	2,407.86	4.00	5.76
Ammonium choride	493.25	1.37	2,096.81	5.82	7.90	529.28	1.47	2,114.51	3.52	6.00
Ammoinium sulfate	514.57	1.43	1,359.37	3.77	8.24	414.71	1.16	2,006.23	3.34	6.20
Yeast extract	536.85	1.49	1,176.66	3.27	8.33	394.28	1.09	1,799.10	3.00	6.40
Peptone	426.17	1.18	1,322.82	3.67	8.53	398.74	1.11	1,849.56	3.10	5.92
Soybean protein	329.68	0.91	1,351.20	3.75	8.48	256.36	0.71	2,627.93	4.38	8.50
Corn steep liquor	287.17	0.98	1,117.73	3.10	7.70	240.58	0.67	1,847.04	3.08	5.62

Culture conditions: Cultivation were carried out at 28°C for 180 rpm.

## Data Availability

The datasets analysed during the current study are not publicly available due to continuing of the doctoral thesis but are available from the corresponding author on reasonable request.
